# Resveratrol ameliorates bisphenol A-induced testicular toxicity in adult male rats: a stereological and functional study

**DOI:** 10.1186/s12610-022-00174-8

**Published:** 2023-01-06

**Authors:** Hossein Bordbar, Seyedeh-Saeedeh Yahyavi, Ali Noorafshan, Elham Aliabadi, Maryam Naseh

**Affiliations:** 1 Histomorphometry and Stereology Research Center, Shiraz University of Medical Sciences, Zand Ave., Shiraz, 71348-45794 Iran; 2 Department of Anatomy, School of Medicine, Shiraz University of Medical Sciences, Shiraz, Iran

**Keywords:** Bisphenol A, Resveratrol, Testicular toxicity, Sperm parameters, Stereology

## Abstract

**Background:**

Bisphenol A (BPA) is one of the most widely used synthetic chemicals worldwide. BPA as an endocrine disruptor affects the reproductive systems through estrogenic and antiandrogenic proprieties. Resveratrol (RES) as a natural polyphenol and potent antioxidant exhibits protective effects against reproductive toxicity by inhibiting of oxidative stress. 48 male rats were divided into eight groups (*n*=6), including CONTROL, OLIVE OIL (0.5 ml/ day), Carboxy methylcellulose (CMC) (1 ml of 10 g/l), RES (100mg/kg/day), low dose of BPA (25 mg/kg/day), high dose of BPA (50 mg/kg/day), low dose of BPA + RES, and high dose of BPA + RES. All treatments were done orally per day for 56 days. At the end of the 8th week, blood samples were collected for hormone assays. Then, the sperm parameters were analyzed, and the left testis was removed for stereological study.

**Results:**

We showed a significant decrease in sperm parameters in the low and high doses of BPA groups compared to control groups (*P*<0.05). The volume of testicular components as well as the diameter and length of seminiferous tubules significantly reduced (11-64 %), and the total number of the testicular cell types decreased (34-67 %) on average in the low and high doses of BPA groups. Moreover, serum follicle-stimulating hormone (FSH), luteinizing hormone (LH), and testosterone hormones concentration showed a significant reduction in both doses of BPA groups (*P*<0.01). Nonetheless, treatment with RES could ameliorate all the above-mentioned changes in the low and high doses of BPA groups (*P*<0.05).

**Conclusions:**

RES could prevent BPA-induced testicular structural changes and sperm quality via improving gonadotropin hormones and testosterone levels.

## Rèsumè

**Contexte:**

Le bisphénol A (BPA) est l’un des produits chimiques synthétiques les plus utilisés dans le monde. Le BPA en tant que perturbateur endocrinien affecte le système reproducteur par le biais de ses propriétés œstrogéniques et anti-androgènes. Le resvératrol (RES), en tant que polyphénol naturel et puissant antioxydant, présente des effets protecteurs contre la toxicité sur la reproduction en inhibant le stress oxydatif. Quarante-huit rats mâles ont été divisés en huit groupes (*n* = 6), comprenant les groupes TÉMOIN, HUILE D’OLIVE (0,5 ml/jour), méthylcellulose Carboxyle (MCC) (1 ml de 10 g/L), RES (100 mg/kg/ jour), faible dose de 25 de BPA (25 mg/kg/jour), dose élevée de BPA (50 mg/kg/jour), faible dose de BPA + RES et dose élevée de BPA + RES. Tous les traitements ont été effectués quotidiennement par voie orale pendant 56 jours. À la fin de la 8ème semaine, des échantillons de sang ont été prélevés pour dosages hormonaux. Ensuite, les paramètres du sperme ont été analysés et le testicule gauche a été retiré pour une étude stéréologique.

**Résultats:**

Nous avons montré une diminution significative des paramètres spermatiques dans les groupes traités par doses faibles et doses élevées de BPA par rapport aux groupe témoin (*P*<0,05). Le volume des composants testiculaires ainsi que le diamètre et la longueur des tubules séminifères ont été considérablement réduits (11-64 %) ; le nombre total des types de cellules testiculaires a diminué (34-67 %) en moyenne dans les groupes traités par doses faibles et doses élevées de BPA. De plus, la concentration sérique d’hormone folliculostimulante (FSH), lutéinisante (LH) et de testostérone a montré une réduction significative dans les groupes traités quelle que soit la dose de BPA (*P*<0,01). Néanmoins, le traitement par RES pourrait améliorer tous les changements mentionnés ci-dessus dans les groupes traités par doses faibles et élevées de BPA (*P*<0,05).

**Conclusions:**

Le RES pourrait avoir un effet positif sur les changements structurels testiculaires induits par le BPA, ainsi que la qualité du sperme, en améliorant les taux sériques d’hormones gonadotrophines et de testostérone.

**Mots-clés:**

Bisphénol A Resvératrol Toxicité testiculaire Paramètres du Sperme Stéréologie

## Background

Bisphenol A (BPA) is one of the most widely used synthetic chemicals worldwide. It is found in large amount of consumer products such as polycarbonate plastics, epoxy resins, linings of cans, medical devices, dental sealants, and many other products that are part of our daily lives [1–3]. Public health has raised concerns about the widespread applications and toxic effects of BPA [4]. Exposure of BPA can occur directly or indirectly through inhalation, dermal exposure and ingestion [5, 6]. It has been reported that the main rout of exposure in humans is oral, which accounts about 90% of BPA exposures. It has been shown that BPA contributes to the cause of several endocrine disorders including reproductive dysfunction, infertility, precocious puberty and hormone dependent tumors [7, 8]. Evidences suggest that BPA exerts the toxic effects on the reproductive system via different mechanisms. BPA as an endocrine disruptor seems to mediate reproductive failure through estrogenic and antiandrogenic proprieties [9]. BPA can interfere with estrogenic signaling pathways by interacting with estrogen receptors (ERs), or by producing a small but potent estrogenic metabolite [10]. BPA can also bind to the androgen receptor (AR) as an antagonist [11], which can disrupt the hypothalamic-pituitary-testicular axis, thereby affecting gene expression and the enzymatic activity of testicular steroidogenesis, leading to hypogonadotropic hypogonadism [12, 13].

In this regard, several animal studies have also confirmed the reproductive toxicity of BPA in rats and mice [14–16]. It has been demonstrated that BPA decreases testis weight, reduces diameter and thickness of seminiferous tubules and leads to compromised spermatogenesis. These morphological alterations and abnormal spermatogenesis seem to be induced by the reduction of reproductive hormone production and promotion of germ cell apoptosis [17, 18]. On the other hand, exposure to BPA is related to the reduced activity of antioxidant enzymes, which could contribute to oxidative stress and sperm damage [19, 20].

Resveratrol (RES; trans-3,5,4’-trihidroxy-trans-stilbene), as a natural polyphenol and potent antioxidant is found in a wide range of foods, especially grapes, berries, and peanuts [21]. Several reports have demonstrated that RES exhibits the protective effects against reproductive toxicity by suppressing lipid peroxidation [22, 23]. Moreover, RES may improve sperm count and motility, as well as decrease germ cell apoptosis by stimulating the hypothalamic–pituitary–gonad axis and enhancing blood testosterone levels [24]. Accordingly, for the first time this study was designed to evaluate the protective effects of RES against deleterious effects of low (25 mg/kg/day) and high doses (50 mg/kg/day) of BPA on the structure and function of testis using stereological assessment, hormonal measurements, and quantitative-qualitative study of sperm parameters.

## Materials and methods

### Animals

Forty eight male Sprague-Dawley rats (age, 6–8 weeks old; weight, 180-210 g) were purchased from the Animal Laboratory Center of Shiraz University of Medical Sciences. The animals were kept under standard conditions at room temperature (22 ± 2 °C), with normal humidity and 12–12 h light-dark cycles. They also had free access to standard food and water. All animal experiments carried out in accordance with the National Institutes of Health guide for the care and use of Laboratory animals (NIH Publications No. 8023, revised 1978). Also, the animal procedures were performed under the standard rules established by the Animal Care and Ethics Committee of Shiraz University of Medical Sciences (IR.SUMS.REC.1398.392).

### Experimental design

**Fig. 1:**
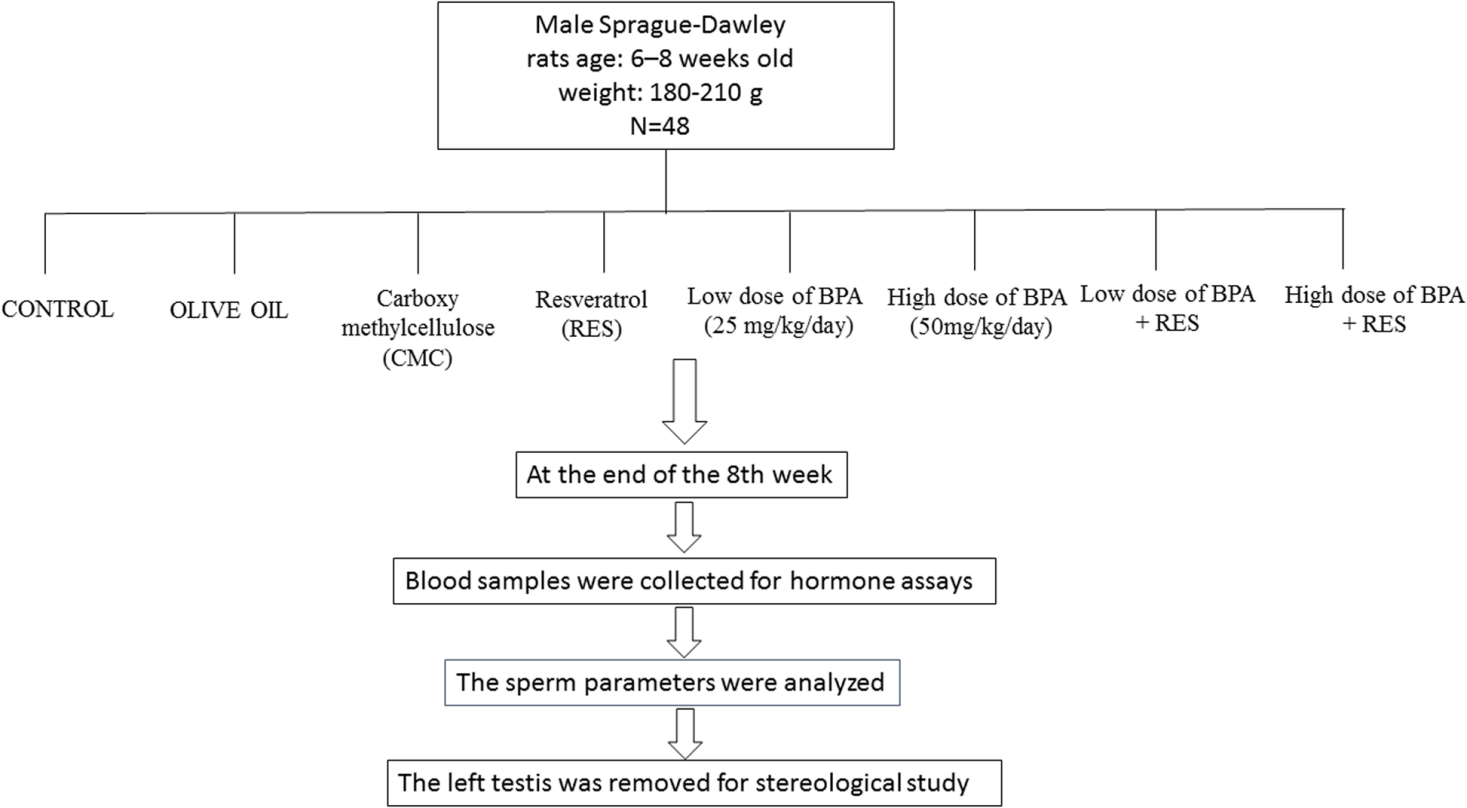
Flow chart of the experimental design

The rats were randomly divided into eight groups (n=6); CONTROL group received distilled water orally per day for 56 days (spermatogenesis length), OLIVE OIL group received 0.5 ml/day Olive oil orally for 56 days, Carboxy methylcellulose (CMC) group received 1 ml of 10 g/l CMC orally [25] per day for 56 days, RES group received 100 mg/kg/day RES that was diluted in CMC and administered orally at a dosing volume of 1 ml [26, 27] for 56 days, BPA-LOW group received low dose of BPA (25 mg/kg/day) orally for 56 days, BPA-HIGH group received high dose of BPA (50 mg/kg/day) [28] for 56 days, BPA was diluted in olive oil and administered daily orally at a dosing volume of 0.5 ml. BPA-LOW + RES group received orally with low dose of BPA plus RES (100mg/kg/day) for 56 days, and BPA-HIGH + RES group received high dose of BPA plus RES (100mg/kg/day) orally for 56 days (Fig. 1).

It should be noted that the dosages of BPA (CAS 80-05-7, Sigma–Aldrich Co., St. Louis, USA) used in current study were based on the previously reported as maximum permissible dose that have no observable side effect on reproductive and developmental toxicity (50 mg/kg BW/day) in rats [13, 29].

### Hormone measurements

At the end of the 8th week (on day 56), fasted rats were killed by cervical dislocation and blood samples were collected from the heart through a cardiac puncture and stored in heparin-free tubes. Then, the samples were centrifuged at 3500 rpm for 15 min. The serum was obtained and stored at -70 °C for subsequent hormone evaluation.

The serum levels of follicle-stimulating hormone (FSH; Category No. CK-30597), luteinizing hormone (LH; Category No. CK-E90904, and testosterone concentrations (Category No. E90243) were determined by rat ELISA kits (From East. Bio Pharm Company) using a microplate reader (Biotek, USA). Briefly, 100 μL of standard or sample was pipetted to each well and incubated for 2 hours at 37 °C. After removing any unbound substances, 100 μL of anti-biotin antibodies was added to the wells. After washing, 100 μL of avidin conjugated Horseradish Peroxidase (HRP) was added to each well and incubated for 1 hour at 37 °C. Then, 90 μL of 3,3'5,5'-Tetramethylbenzidine (TMB) substrate was added to each well and incubated for 20 minutes at 37 °C. Finally, the color development was stopped and the absorbance was determined at 450 nm using a microplate reader.

### Spermatozoa counts, morphology and motility

Immediately after blood collection, the proximal part of the vas deferens just distal to the cauda epididymis (10 mm) was removed, and moved to a petri dish containing 3 mL normal saline solution. The suspension was gently shaken at 37°C for 5-10 min to diffuse the spermatozoa. The samples were counted in a hemocytometer. Ten fields were then randomly selected and evaluated for motility grading to distinguish the immotile sperms from those with progressive or non-progressive motility. Also, the sperm smears were stained with 1% eosin Y for assessing the morphology [30].

There is two types of progressive motility: 1- rapid progressive motility, 2- slow progressive motility. The efficient passage of spermatozoa through cervical mucus is dependent on rapid progressive motility.

We should add that it is necessary to distinguish between these two types of progressive motility. So that neglecting the distinction between two progressive sperm groups leads to ignoring the information in the semen sample, and the removal of such useful information would impoverish the semen analysis [31].

### Stereological study

The left testis was removed and weighed. Then, according to the immersion method, it was immersed in isotonic saline-filled jar for measuring the primary volume “V (testicle)” [32]. Afterwards, the samples were fixed in 4% buffered formaldehyde solution for stereological studies. The orientator method was applied to obtain Isotropic Uniform Random (IUR) sections [32]. About 8-12 slabs in each testis were collected through this procedure. To estimate the shrinkage, a circle was punched out from a random testis slab by a trocar (diameter 5 mm), and the trocar radius was considered as the “area (before)” (πr2). After tissue processing, the area was calculated as the “area (after)”. After tissue processing and paraffin embedding, 5 and 25 μm sections were cut by the microtome and were stained using Hematoxylin-Eosin (H&E). The areas of the circles were measured before processing (unshrunk) and after processing (shrunk) and finally, the degree of shrinkage “d (shr)” was calculated by the following formula:

$$\mathrm d(\mathrm{shr})=1-{\lbrack\mathrm{Area}(\mathrm{after})/\mathrm{Area}(\mathrm{before})\rbrack}^{1.5}$$

Then, the total volume of the testis was evaluated with regard to tissue shrinkage [V(shrunk)] using the following formula:

$$\mathrm V(\mathrm{shrunken})=\mathrm V(\mathrm{unshrunk})\times\lbrack1-\mathrm d(\mathrm{shr})\rbrack$$

#### Estimation of the testicular components volume

The volume density of the testis sections was analyzed by a video microscopy system. In doing so, the point grid was superimposed on the microscopic images of the H&E-stained sections (5μm thickness) on a monitor by the software designed at the Histomorphometry and Stereology Research Center. The volume density “Vv (structure/testis)” of the testicular components, including seminiferous tubules, interstitial tissue, and germinal epithelium, was estimated by the point counting method [33, 34]. Finally, the total volume of each component was obtained by the following formula:

$$\mathrm V(\mathrm{structure})=\mathrm{Vv}(\mathrm{structure}/\mathrm{testis})\times\mathrm V(\mathrm{shrunk})$$

#### Estimation of the length and diameter of seminiferous tubules

The length density (Lv) of the seminiferous tubules was measured on the sampled tubules in an unbiased counting frame applied on the 5 μm thick sections (H&E staining) [35], and calculated by the following formula:

$$\mathrm{Lv}=2\Sigma\mathrm{Q}/\lbrack\Sigma\mathrm{P}\times(\mathrm a/\mathrm f)\rbrack$$

Where “Σ*Q*” is the total number of the selected tubules, “ΣP” represents the total points superimposed on the testis, and “a/f*”* indicates the area of the counting frame. The total length of the seminiferous tubules “L(tubules)” was calculated by multiplying the lengths density (Lv) by V(structure) [36].

$$\mathrm L(\mathrm{tubules})=\mathrm{Lv}\times\mathrm V(\mathrm{structure})$$

The diameter of the seminiferous tubules was also measured on the sampled tubules in the counting frame. The diameter was measured perpendicularly to the long axis of the tubules where the tubules were widest [35]. An average of 100 tubules were counted per testis.

#### Estimation of number of testicular cell types

A computer linked to a light microscope (Nikon E200, Japan) with 40× oil lens (NA=1.4) was used to assess the total number of testicular cell types, including spermatogonia (A and B), spermatocytes, round spermatids (steps 1–8 spermiogenesis), long spermatids (steps 9–16 spermiogenesis), Sertoli and Leydig cells.

The total number of the testicular cell types was calculated using the optical disector method applied on the H&E-stained sections (25μm thickness) [37]. In so doing, the microscopic fields were scanned by moving the microscope stage at equal distances in X and Y directions based on systematic uniform random sampling. The movement in Z direction was also performed using a microcator (MT12, Heidenhain, Germany) fixed on the microscope stage. The Z-axis distribution from the sampled cells in different focal planes was plotted to determine the guard zones and disector’s height [38]. The numerical density (Nv) was estimated using the following formula:

$$\mathrm{Nv}=\Sigma\mathrm{Q}/(\Sigma\mathrm{A}\times\mathrm h)\times(\mathrm t/\mathrm{BA})$$

Where “ΣQ” was the number of each cell type nuclei coming into focus, “ΣA” indicated the total area of the unbiased counting frame, “h” represented the disector’s height, “t” was the mean section thickness, and “BA” was the microtome block advance. Finally, the total number of the testicular cell types was calculated by multiplying the numerical density (Nv) by V(structure):

$$\mathrm N(\mathrm{cells})=\mathrm{Nv}\times\mathrm V(\mathrm{structure})$$

Where, V(structure) was the total volume of the germinal epithelium for the germinal layer cells and the total volume of the interstitial tissue for the Leydig cells.

### Statistical analysis

The data were expressed as mean ± standard error (SEM). The results were analyzed by one-way analysis of variance (ANOVA) and Tukey’s post hoc test using Graph Pad Prism 6 software (San Diego, CA, USA). *P*<0.05 was considered to be statistically significant.

## Results

### Spermatozoa count, normal morphology and motility

According to Table 1, a significant decrease was observed in the count, percentage of normal morphology, and motility of spermatozoa in the rats exposed to low and high doses of BPA groups compared to control group (*P*<0.05 and *P*<0.01, respectively). However, these parameters in the BPA-LOW + RES and BPA-HIGH + RES groups improved compared to the BPA groups (*P*<0.01 and *P*<0.05, respectively).

**Table 1:** Comparison of sperm parameters. Mean ± SEM of the Count (×106), Normal morphology (%), Motility (%), and Immotile (%) in the CONTROL, OLIVE OIL, carboxy methylcellulose (CMC), resveratrol (RES), low dose of Bisphenol A (BPA-LOW), high dose of BPA (BPA-HIGH), BPA-LOW + RES, and BPA-HIGH + RES groups. *n* = 6 in each group. The results were analyzed by one-way analysis of variance (ANOVA) and Tukey’s post hoc test. * *P*<0.05, ** *P*<0.01 vs. CONTROL; ## *P*<0.01 vs. BPA-LOW; $ *P*<0.05 vs. BPA-HIGH

Groups	Count (×10^**6**^)	Normal morphology (%)	Motility (%)			
			Rapid progressive	Slow progressive	Non progressive	Immotile
CONTROL	6.08±1.8	89.6±5.08	34.6±4.5	20.8±3.2	21.1±2.4	21.3±6.2
OLIVE OIL	6.12±2.3	85.6±3.18	35.4±1.6	21.4±2.3	21.1±6.2	21.1±6.4
CMC	5.8±3.2	81.6±2.5	32.0±2.41	23.0±2.6	23.4±5.5	22.3±1.9
RES	6.52±2.2	91.5±3.05	36.7±6.4	22.0±3.8	21.6±7.7	19.7±4.2
BPA-LOW	3.21±1.08*	54.9±10*	28.2±2.9*	24.4±3.1*	25.1±3.3*	26.3±2.7*
BPA-HIGH	2.63±1.4**	30.9±10**	23.2±2.5**	29.6±2.4**	30.2±2.3**	32.7±2.5**
BPA-LOW+RES	5.22±.99^**##**^	67.7±15.2^**##**^	33.5±1.6^**##**^	26.4±2.6^**##**^	24.4±5.5	26.7±1.9^**##**^
BPA-HIGH+RES	3.76± 1.9^$^	44.5±8.2^**$**^	29.1±2.7^**$**^	25.3±2.1^**$**^	26.4±3	27.2±1.2^**$**^

### Qualitative changes

**Fig. 2:**
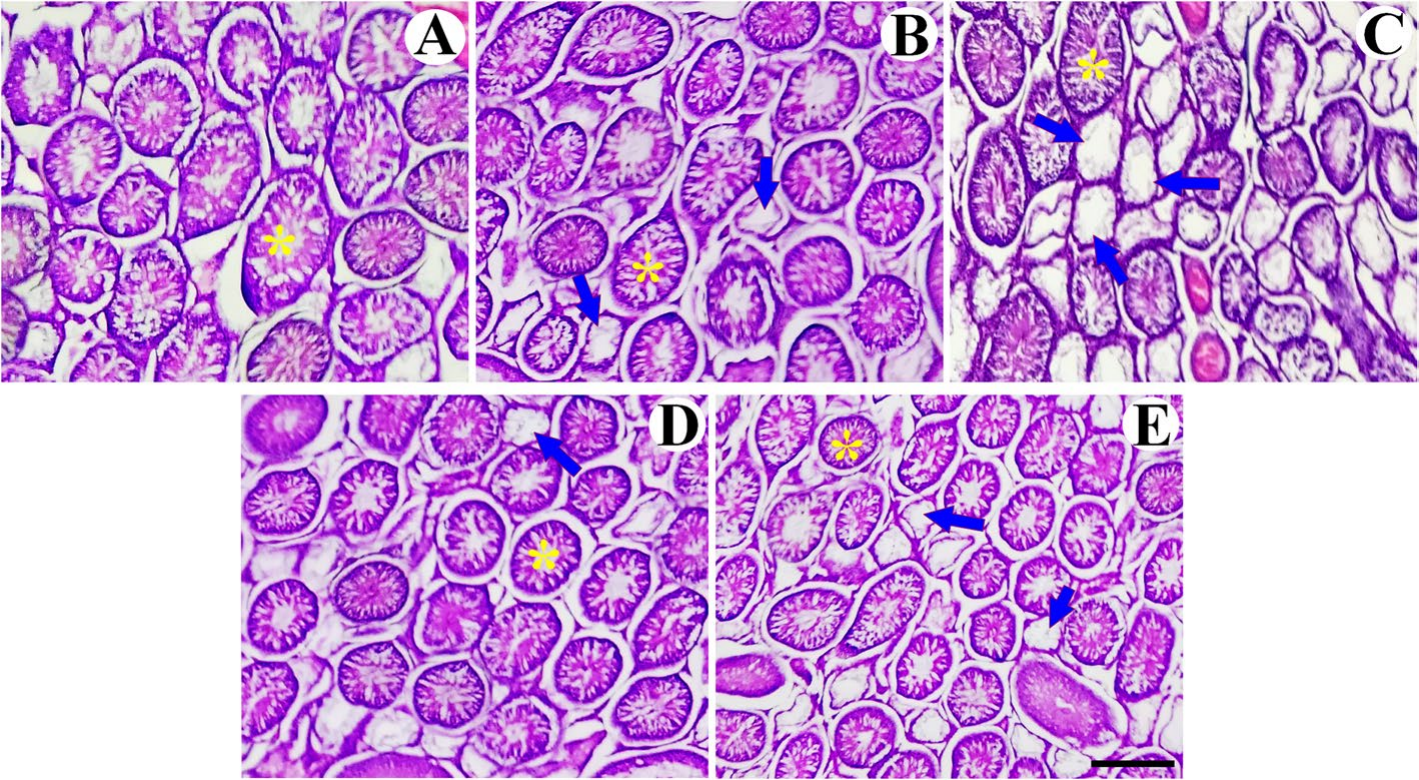
Testicular histological evaluation. Representative photomicrographs of testis sections stained with hematoxylin & eosin (H&E) in the CONTROL (**A**), low dose of Bisphenol A (BPA-LOW) (**B**), high dose of BPA (BPA-HIGH) (**C**), BPA-LOW + resveratrol (RES) (**D**), and BPA-HIGH + RES (**E**) groups. All plates are to the same scale (Scale bar = 200 μm). *The images* indicate the normal seminiferous tubules (asterisk), and atrophied seminiferous tubules (arrow)

Qualitative evaluation of the testis has been presented in Fig. 2. The histological sections of the low and high doses of BPA rats showed the structural changes, including atrophy and reduced number of seminiferous tubules. Concomitant treatment of these groups with RES ameliorated these destructive effects.

### Stereological assays

#### The volume of the testicle

**Fig. 3:**
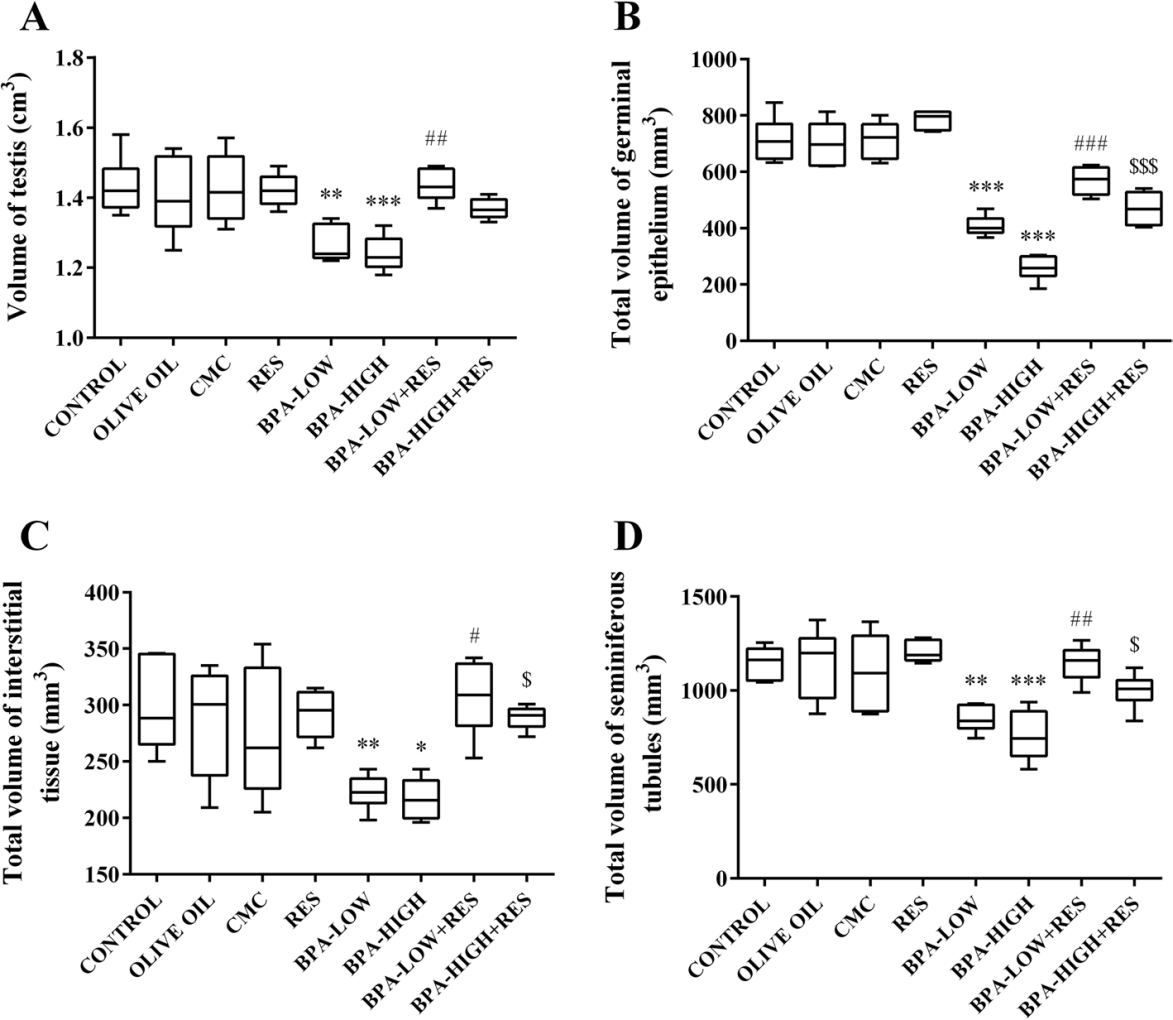
The evaluation of volume. The box plots represents the volume of the testis (**A**), germinal epithelium (**B**), interstitial tissue (**C**), and seminiferous tubules (**D**) in the CONTROL, OLIVE OIL, carboxy methylcellulose (CMC), resveratrol (RES), low dose of Bisphenol A (BPA-LOW), high dose of BPA (BPA-HIGH), BPA-LOW + RES, and BPA-HIGH + RES groups. *n* = 6 in each group. The results were analyzed by one-way analysis of variance (ANOVA) and Tukey’s post hoc test. Data are presented as mean ± SEM. *p < 0.05, **p < 0.01, and ***p < 0.001 vs. CONTROL; #*p* < 0.05, ##*p* < 0.01, and ###*p* < 0.001 vs. BPA-LOW; $*p* < 0.05 and $$$*p* < 0.001 vs. BPA-HIGH

The results showed a significant reduction in the testicle volume by 11.7 % and 13.5 % in the rats exposed to low and high doses of BPA compared to the control groups, respectively (*P*<0.01 and *P*<0.001). However, the testis volume recovered considerably in the animals that received BPA-LOW + RES group compared to BPA-LOW group (*P*<0.01) (Fig. 3A).

#### The volume of germinal epithelium

The total epithelial volume in rats treated with low and high doses of BPA decreased 43% and 64% in comparison to the control groups, respectively (*P*<0.001). Treatment with RES ameliorated the epithelial volume changes in the low or high doses of BPA groups (*P*<0.001) (Fig. 3B).

#### The volume of interstitial tissue

The results indicated that the interstitial tissue volume reduced 25.3% and 27.3% in the low and high doses of compared to the control groups, respectively (*P*<0.01 and *P*<0.05). However, this parameter significantly was increased in the rats treated with RES in the low or high doses of BPA groups (*P*<0.05) (Fig. 3C).

#### The volume of seminiferous tubules

A significant reduction was seen in the total volume of seminiferous tubules by 26.2% and 34% in the low and high doses of BPA compared to the control groups, respectively (*P*<0.01 and *P*<0.001). Nevertheless, seminiferous volume significantly was ameliorated in the BPA-LOW + RES and BPA-HIGH + RES groups compared to the BPA groups (*P*<0.01 and *P*<0.05, respectively) (Fig. 3D).

#### Diameter of the seminiferous tubules

**Fig. 4:**
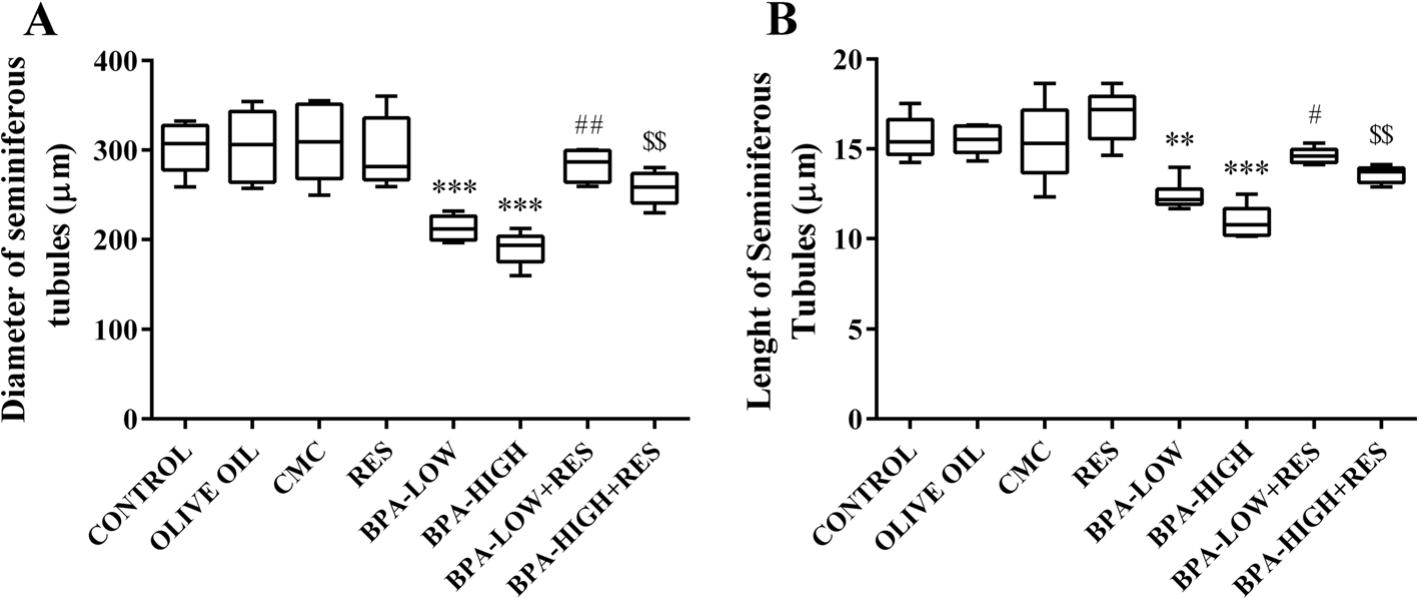
The evaluation of diameter and length of seminiferous tubules. The box plots shows the diameter (**A**), and the length (**B**) of seminiferous tubules in the CONTROL, OLIVE OIL, carboxy methylcellulose (CMC), resveratrol (RES), low dose of Bisphenol A (BPA-LOW), high dose of BPA (BPA-HIGH), BPA-LOW + RES, and BPA-HIGH + RES groups. *n* = 6 in each group. The results were analyzed by one-way analysis of variance (ANOVA) and Tukey’s post hoc test. Data are presented as mean ± SEM. **p < 0.01, and ***p < 0.001 vs. CONTROL; #*p* < 0.05, ##*p* < 0.01vs. BPA-LOW; $$*p* < 0.01 vs. BPA-HIGH

The diameter of the seminiferous tubules decreased 29.7% and 37.3% in rats treated with low and high doses of BPA compared to the control group (*P*<0.001). Treatment with RES increased this parameter in the low and high doses of BPA groups (*P*<0.01) (Fig. 4A).

#### Length of the seminiferous tubules

The results showed that length of the seminiferous tubules have reduced 20.6% and 29.8% in the low and high doses of BPA compared to the control groups (*P*<0.01 and *P*<0.001, respectively). Nonetheless, the tubules length significantly was improved in the rats treated with RES in the low and high doses of BPA groups (*P*<0.05 and *P*<0.01, respectively) (Fig. 4A).

#### Number of spermatogonia A and B

**Fig. 5:**
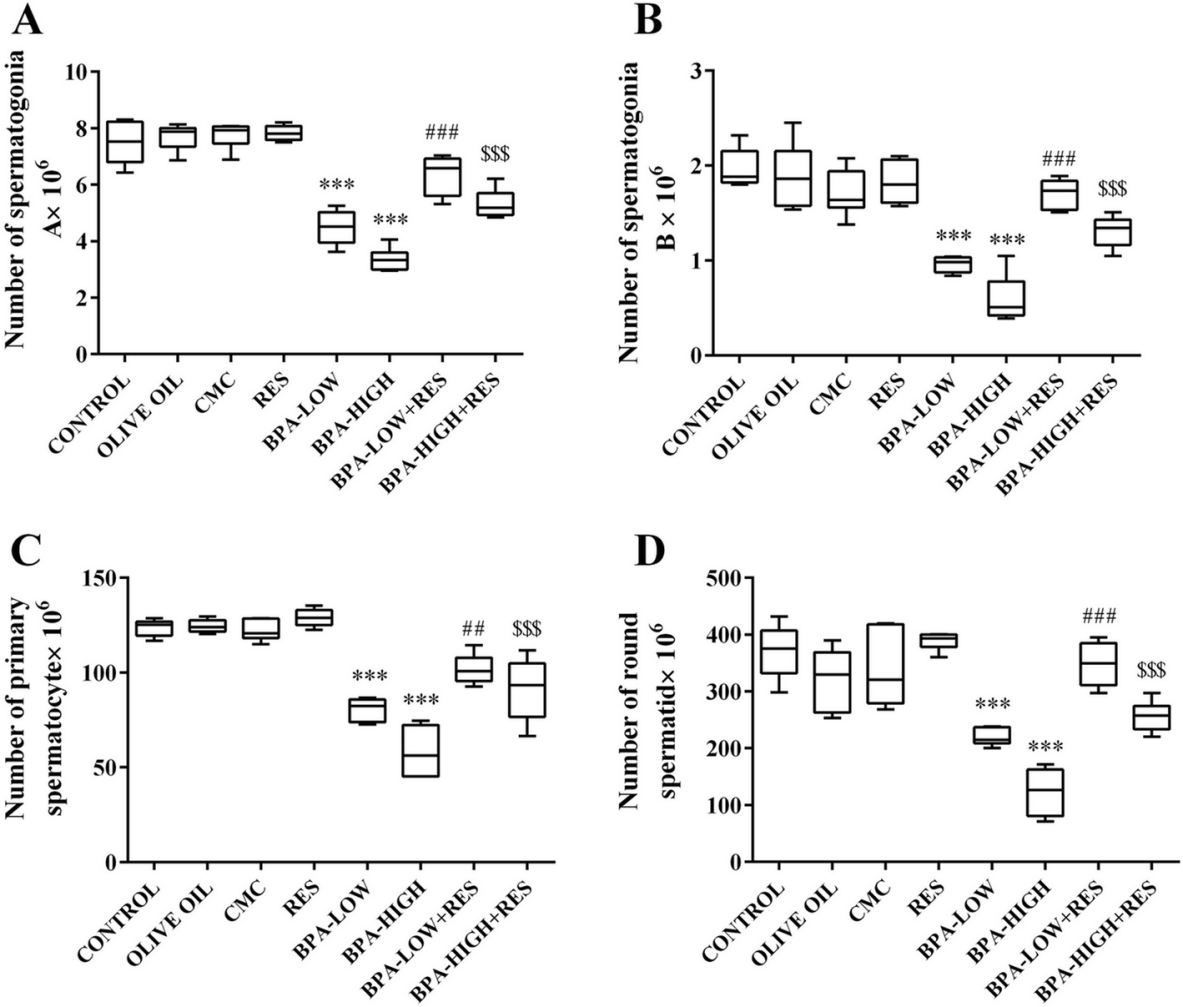
Evaluation of the germinal cells number. The box plots represents the number of spermatogonia A (**A**), spermatogonia B (**B**), spermatocytes (**C**), and round spermatids (**D**) in the CONTROL, OLIVE OIL, carboxy methylcellulose (CMC), resveratrol (RES), low dose of Bisphenol A (BPA-LOW), high dose of BPA (BPA-HIGH), BPA-LOW + RES, and BPA-HIGH + RES groups. The results were analyzed by one-way analysis of variance (ANOVA) and Tukey’s post hoc test. Data are presented as mean ± SEM. ***p < 0.001 vs. CONTROL; #*p* < 0.05, ##*p* < 0.01, and ###*p* < 0.001 vs. BPA-LOW; $$*p* < 0.01 and $$$*p* < 0.001 vs. BPA-HIGH

The total number of spermatogonia A reduced by 40.03% and 55.2%, and spermatogonia B by 51.27% and 70.05% in the low and high doses of BPA compared to the control groups, respectively (*P*<0.001). However, treatment with RES increased these cells in the low and high doses of BPA groups (*P*<0.001) (Fig. 5A and B).

#### Number of spermatocytes

Statistical analysis showed 34.85% and 53% reduction in the number of spermatocytes for both the low and high doses of BPA compared to the control groups (*P*<0.001). Treatment with RES ameliorated these changes in the BPA-LOW + RES and BPA-HIGH + RES groups compared to the BPA groups (*P*<0.01 and *P*<0.001, respectively) (Fig. 5C).

#### Number of round and long spermatid

**Fig. 6:**
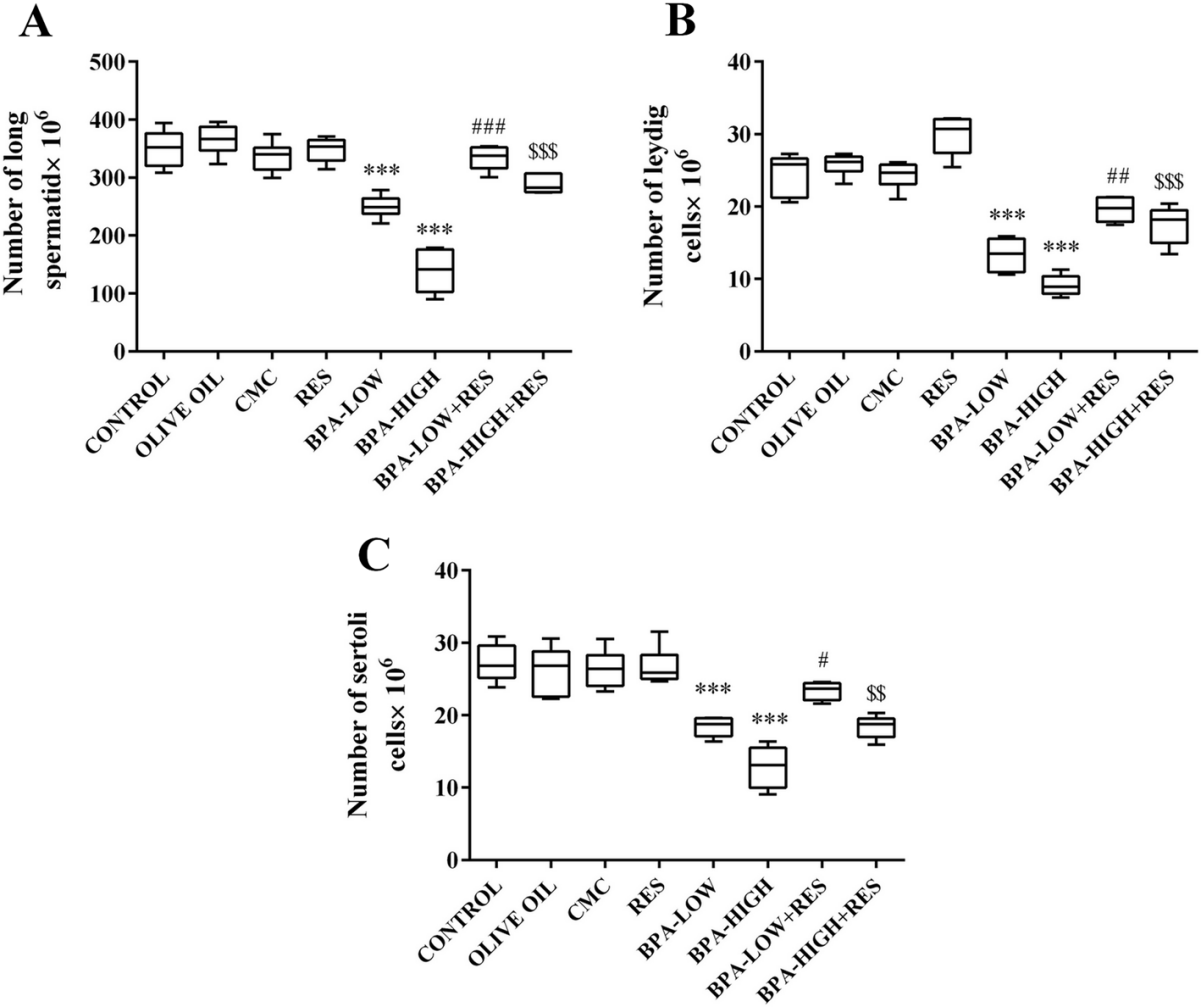
Evaluation of long spermatids, Leydig and Sertoli cells number. The box plots represents the number of long spermatids (**A**), Leydig (**B**), and Sertoli (**C**) in the CONTROL, OLIVE OIL, carboxy methylcellulose (CMC), resveratrol (RES), low dose of Bisphenol A (BPA-LOW), high dose of BPA (BPA-HIGH), BPA-LOW + RES, and BPA-HIGH + RES groups. The results were analyzed by one-way analysis of variance (ANOVA) and Tukey’s post hoc test. Data are presented as mean ± SEM. ***p < 0.001 vs. CONTROL; #*p* < 0.05, ##*p* < 0.01, and ###*p* < 0.001 vs. BPA-LOW; $$*p* < 0.01 and $$$*p* < 0.001 vs. BPA-HIGH

The number of round spermatids decreased by 40.76% and 66.72%, and long spermatids by 28.7% and 60.35% in the low and high doses of BPA, respectively compared to the control groups (*P*<0.001). Moreover, ameliorative effects of RES on the number of these cells were seen in rats treated with low and high doses of BPA groups (*P*<0.001) (Figs. 5D and 6A).

#### Number of Leyding and Sertoli cells

A significant reduction was seen in the number of Leydig cells by 45.78% and 62.85%, and Sertoli cells by 32.28% and 52.76% in the low and high doses of BPA than those of the control groups, respectively (*P*<0.001). Treatment with RES recovered the number of Leydig cells and Sertoli cells in the BPA-LOW + RES (*P*<0.01 and *P*<0.05, respectively), and BPA-HIGH + RES (*P*<0.001 and *P*<0.01, respectively) groups compared to the BPA groups (Fig. 6B and C)

#### Hormone assays

**Fig. 7:**
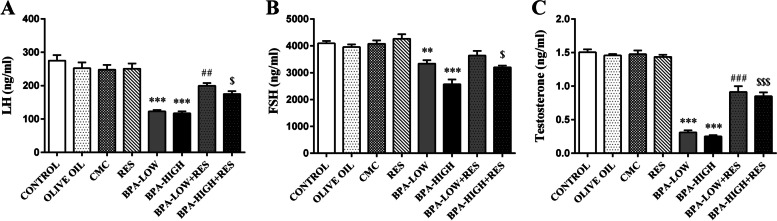
Serum concentrations of luteinizing hormone (LH), follicle-stimulating hormone (FSH), and testosterone hormones. The column graphs represent the concentrations of LH (**A**), FSH (**B**), and testosterone (**C**) in the CONTROL, OLIVE OIL, carboxy methylcellulose (CMC), resveratrol (RES), low dose of Bisphenol A (BPA-LOW), high dose of BPA (BPA-HIGH), BPA-LOW + RES, and BPA-HIGH + RES groups. *n* = 6 in each group. The results were analyzed by one-way analysis of variance (ANOVA) and Tukey’s post hoc test. Data are presented as mean ± SEM. **p < 0.01, and ***p < 0.001 vs. CONTROL; #*p* < 0.05, ##*p* < 0.01vs. BPA-LOW; $$*p* < 0.01 vs. BPA-HIGH

The gonadotropins assessment showed a significant reduction in serum LH and FSH levels in the BPA-LOW (*P*<0.001 and *P*<0.01, respectively), and BPA-HIGH (*P*<0.001) groups compared to the control group. Also, the testosterone concentration of the rats given low or high doses of BPA was lower than in the control group (*P*<0.001). The RES exposure led to significant increase in the serum LH and testosterone levels in the BPA-LOW (*P*<0.01 and *P*<0.001, respectively), and BPA-HIGH (*P*<0.05 and *P*<0.001, respectively) groups, while the serum FSH levels significantly increased only in the BPA-HIGH + RES group (*P*<0.05) (Fig. 7).

## Discussion

The current study revealed the ameliorative effects of RES on testicular damage induced by BPA in rats. The first part of our findings showed the deleterious effects of two doses of BPA, 25 and 50 mg/kg/day for 8 weeks, on sperm quality and structural changes of the testis. The earlier studies showed that 50 mg/kg/day is considered as maximum permissible dose that have no observable side effect on reproductive and developmental toxicity [39]. But we found that ingestion of BPA at these dosages had adverse effects on count, morphology, and motility of spermatozoa. In line with our results, it has been observed a reduction in epididymal sperm motility and count in the rats exposed to BPA at the 10 and 50 mg/kg in a dose dependent manner [40]. Also, it has been demonstrated that BPA at 5 and 25 mg/kg/day reduced sperm production, reserves and transit time through the epididymis [13]. Moreover, long-term exposure to 0.2 mg/kg BPA in rats led to decreased sperm count and inhibited spermiation [41].

The reduction in sperm count and quality is in accord with decreased stereological parameters. The changes of structural indices including volume, diameter and length of seminiferous tubules suggest the atrophy of these tubules and testicular abnormalities due to BPA. Loss of the germinal epithelial cells was also seen after exposure to both doses of BPA. A reduction in germinal epithelial volume could be a consequence of decline in the number of germinal cells. The reduction in sperm production could be related to the disruption of spermatogenesis. Jin et al., 2013 also reported that BPA exposure could decrease sperm count via the reduction in type A spermatogonial, spermatocytes and spermatids. BPA impaired spermatogenesis through suppressing reproductive hormones and activating germ cells apoptosis mediated by Fas/FasL signaling pathway [42, 43]. Sertoli cells are another type of cells in the seminiferous tubules, which have a supportive and nutrient function. Since, Sertoli cells can affect the proliferation and differentiation of germinal cells, and also help in the process of spermatogenesis. So, it seems the loss of these supporting cells could be led to deficiency of supportive functions in BPA-treated rat, and cause the loss of spermatogenic cells. It has been indicated that Sertoli cells are targets of pituitary-derived FSH and testosterone to transduce signals into paracrine regulation of spermatogenesis [44–46]. Accordingly, Sertoli cell depletion following BPA treatment in present study may be due to a decrease in FSH and testosterone levels. On the other hand, testosterone secretion is produced in Leydig cells of testicular interstitium in response to LH [47]. Therefore, the lack of LH stimulation in BPA-treated groups could justify the reduction of Leydig cells and interstitial tissue atrophy and also the decrease of testosterone production. Testosterone is an essential hormone to maintain normal spermatogenesis and prevention of germ cell apoptosis in adult rats [48]. So, it is reasonable to assume that the inhibition of reproductive hormones production may have contributed to spermatogenesis impairment induced by BPA. Similarly, BPA could cause defective spermatozoa by disruption of the hypothalamic–pituitary–gonadal axis, causing a state of hypogonadotropic hypogonadism [13, 42, 49]. Another possible hypothesis may be involved in spermatogenesis dysfunction is the effects of BPA-induced oxidative damage. BPA exposure could induce ROS production by reducing the activity of the antioxidant system [50]. The adverse effects of BPA on sperm count and quality due to oxidative stress have been described by previous studies [40, 49].

The second step of our study demonstrated the protective effects of RES against BPA- induced testicular structural changes and sperm quality. Our results indicated that the concomitant treatment of the BPA groups with RES for 8 weeks could significantly restore the sperm parameters and prevent testicular atrophy and apoptosis of the testicular cell types. Furthermore, RES enhanced testosterone, FSH, and LH levels in BPA groups. The improvement of testicular structural and sperm quality seems to be related to increased gonadotropin hormones and testosterone levels. Consistent with these findings, previous reports have also shown that the levels of FSH, LH, and testosterone increased in the cisplatin+RES-treated rats compared to cisplatin group, thereby improving sperm parameters and testicular apoptosis [24]. Also, they showed that RES enhanced hormonal levels as well as sperm motility and count compared to control group. But in our study, there was no significant difference between the RES and control groups. The difference between the results of our study and Shatti's research may be because of different route of administration and dose of RES [24].

Also, another earlier study claimed that RES could ameliorate negative effects against BPA-induced reproductive toxicity in mice via reducing oxidative stress [51]. Our results support the contribution of reproductive hormones in the ameliorative effects of RES on BPA-induced testicular toxicity in rats. Meanwhile, a reduction of oxidative stress, as shown by other studies [52], may also be involved in RES protective effects, which required further studies to be confirmed.

One of the limitations of our study was that the signaling pathways that contribute to the amelioration of reproductive hormones by effect on the hypothalamic–pituitary–gonadal axis in the RES treated rats following BPA, which led to spermatogenesis improvement were not investigated.

## Conclusion

In conclusion, the present study demonstrated the protective effects of RES against BPA-induced testicular structural changes and sperm quality via improving gonadotropin hormones and testosterone levels.

## Data Availability

The datasets used and/or analyzed during the current study are available from the corresponding author on reasonable request.

## Abbreviations

BPA: Bisphenol A
RES: Resveratrol
CMC: Carboxy methylcellulose
LH: Luteinizing hormone
FSH: Follicle-stimulating hormone
ER: Estrogen receptor
AR: Androgen receptor
